# Cervical sensorimotor control in idiopathic cervical dystonia: A cross‐sectional study

**DOI:** 10.1002/brb3.735

**Published:** 2017-08-11

**Authors:** Joke De Pauw, Rudy Mercelis, Ann Hallemans, Sarah Michiels, Steven Truijen, Patrick Cras, Willem De Hertogh

**Affiliations:** ^1^ Department of Physical Therapy and Rehabilitation Sciences University of Antwerp Wilrijk Belgium; ^2^ Department of Neurology Antwerp University Hospital Wilrijk Belgium; ^3^ Multidisciplinary Motor Centre Antwerp (M^2^OCEAN) Wilrijk Belgium; ^4^ Faculty of Medicine and Health Sciences Born Bunge Institute University of Antwerp Wilrijk Belgium

**Keywords:** dystonia, position sense, proprioception, sensorimotor control, spasmodic torticollis

## Abstract

**Objectives:**

Patients with idiopathic adult‐onset cervical dystonia (CD) experience an abnormal head posture and involuntary muscle contractions. Although the exact areas affected in the central nervous system remain uncertain, impaired functions in systems stabilizing the head and neck are apparent such as the somatosensory and sensorimotor integration systems. The aim of the study is to investigate cervical sensorimotor control dysfunction in patients with CD.

**Material and Methods:**

Cervical sensorimotor control was assessed by a head repositioning task in 24 patients with CD and 70 asymptomatic controls. Blindfolded participants were asked to reposition their head to a previously memorized neutral head position (NHP) following an active movement (flexion, extension, left, and right rotation). The repositioning error (joint position error, JPE) was registered via 3D motion analysis with an eight‐camera infrared system (VICON ^®^ T10). Disease‐specific characteristics of all patients were obtained via the Tsui scale, Cervical Dystonia Impact Profile (CDIP‐58), and Toronto Western Spasmodic Rating Scale.

**Results:**

Patients with CD showed larger JPE than controls (mean difference of 1.5°, *p *<* *.006), and systematically ‘overshoot’, i.e. surpassed the NHP, whereas control subjects ‘undershoot’, i.e. fall behind the NHP. The JPE did not correlate with disease‐specific characteristics.

**Conclusions:**

Cervical sensorimotor control is impaired in patients with CD. As cervical sensorimotor control can be trained, this might be a potential treatment option for therapy, adjuvant to botulinum toxin injections.

## 1 INTRODUCTION

Adult‐onset idiopathic cervical dystonia (CD) is a movement disorder which is characterized by sustained or intermittent muscle contractions causing abnormal, often repetitive movements, postures, or both. Dystonic movements are typically patterned, twisting and may be tremulous and is frequently accompanied by pain (Albanese et al., 2011; Jinnah & Albanese, 2014). It is the most common form of focal dystonia with a prevalence in Europe ranging from 44 to 183 cases per million (Defazio, Jankovic, Giel, & Papapetropoulos, 2013). Treatment of choice is injections with botulinum toxin (Albanese et al., 2011) and physical therapy is often used as an adjuvant therapy (De Pauw et al., 2014).

The cause of CD remains unknown but in addition to motor symptoms CD is associated with nonmotor symptoms such as sensory deficits, sleep disorders, and pain (Avanzino et al., 2010; Antelmi et al., 2016; Tinazzi, Fiorio, Fiaschi, Rothwell, & Bhatia, 2009; Patel, Jankovic, & Hallett, 2014). Impaired sensorimotor integration has been described as part of the pathophysiology of CD (Tinazzi, Rosso, & Fiaschi, 2003; Fiorio et al., 2007; Konczak & Abbruzzese, 2013). In this paper, we focus on cervical sensorimotor control. Sensorimotor control is a feedback and feedforward mechanism which incorporates all afferent and efferent sensory information (i.e. visual, vestibular and somatosensory input) for central integration and processing to produce motor program execution. The high density of muscle spindles in the suboccipital muscles (Kulkarni, Chandy, & Baby, 2001) and the presence of mechanoreceptors in cervical facet joints (McLain, 1994) contribute in providing cervical afference. Adequate neck proprioception is important to keep the head upright (Shaikh, Wong, Zee, & Jinnah, 2013; Anastasopoulos et al., 1998) and to maintain posture and balance (Mergner, Nasios, Maurer, & Becker, 2001; Treleaven, 2008). Previous investigation of cervical proprioception in patients with CD shows abnormal perception of movement induced by muscle vibration (Bove, Brichetto, Abbruzzese, Marchese, & Schieppati, 2004; Lekhel et al., 1997; Grünewald, Yoneda, Shipman, & Sagar, 1997), abnormal sensory processing in both somatosensory cortices (Molloy, Carr, Zeuner, Dambrosia, & Hallett, 2003), and impaired reflex–voluntary interactions involving neck proprioceptive reflexes (Anastasopoulos, Maurer, & Mergner, 2014). It is, however, unclear to what extend these laboratory induced alterations in neck proprioception are present during voluntary neck movements and cervical sensorimotor control. As deficiencies in cervical sensorimotor control may lead to disturbances in balance, posture, and neck pain (Treleaven, 2008; Revel, Andre‐Deshays, & Minguet, 1991; Treleaven, Jull, & Sterling, 2003; Treleaven, Jull, & LowChoy, 2006; Eva‐Maj, Hans, Per‐Anders, Mikael, & Måns, 2013; Heikkilä & Wenngren, 1998), this could reinforce abnormal neck movements and head posture in CD.

Cervical sensorimotor control is clinically evaluated by head repositioning tasks. The most valid and reliable assessment is the “head repositioning accuracy (HRA) test” to a previously defined neutral head position (NHP) (Michiels et al., 2013). It measures cervical sensorimotor control by evaluating the ability to relocate the head to the memorized NHP after performing an active movement without any visual input. It is expressed by joint position error (JPE) in degrees (°) (Revel et al., 1991; Michiels et al., 2013). The HRA test can discriminate patients from healthy controls (Michiels et al., 2013; Kristjansson, Dall'Alba, & Jull, 2003) and has previously been used in patients populations with impaired cervical sensorimotor control such as whiplash‐associated disorders (Treleaven et al., 2003; Heikkilä & Wenngren, 1998; Treleaven et al., 2008), dizziness (Treleaven et al., 2003; Heikkilä & Wenngren, 1998; Hill et al., 2009) and cervicogenic headache (De Hertogh et al., 2008; Jull, Amiri, Bullock‐Saxton, Darnell, & Lander, 2007). Rehabilitation programs training cervical sensorimotor control resulted in clinical improvement of patients with neck complaints affecting cervical sensorimotor control (Revel, Minguet, Gregoy, & Vaillant, 1994; Jull, Falla, Treleaven, Hodges, & Vicenzino, 2006; Chiarotto, Fortunato, & Falla, 2015). If cervical sensorimotor control is impaired in patients with CD, training hereof might be a complementary treatment option. Therefore, we aimed to investigate whether cervical sensorimotor control is impaired in patients with idiopathic adult‐onset CD.

## 2 MATERIAL AND METHODS

### 2.1 Setting and participants

A cross‐sectional study was conducted. Fifty‐one consecutive patients diagnosed with adult‐onset idiopathic CD were contacted at a tertiary care center in the department of Neurology at the Antwerp University Hospital. In total, 24 of the 51 patients agreed to participate. Main reasons for nonparticipation were lack of time (*n *=* *7), no interest in the study (*n *=* *3), or lack of informed consent (*n *=* *16). All patients were diagnosed by an experienced neurologist in accordance with the European Federation of Neurological Societies/Movement Disorders Society European Section (EFNS/MDS‐ES) guidelines (Albanese et al., 2011) and received regular treatments of botulinum toxin injections. The assessment took place at least 3 months after the last injection, immediately prior to a new injection of botulinum toxin when the clinical effect of the injection was no longer present. Patients were excluded in case of clinical features suggestive for segmental distribution of dystonia, other neurological disorders, vestibular dysfunction, or previous surgery of the cervical spine and alcohol intake in the past 24 hr.

Data from a normative database were used (*n *=* *70). These asymptomatic controls had no bothersome neck pain in the past 6 months, neck or head trauma in the past 5 years, previous surgery or fracture of the cervical spine, rheumatoid arthritis, neurological or vestibular dysfunction, and alcohol intake in the past 24 hr. The group consisted of at least 10 participants per decade (30–90 years), except for decade +80 years (*n *=* *4).

The protocol was approved by the Ethics Committee of the Antwerp University Hospital (reference 14/8/74) and all participants provided informed consent. Recruitment took place from August 2014 to November 2015 and tests were performed in the Multidisciplinary Motor Centre Antwerp (M^2^OCEAN).

### 2.2 Test procedure

The following disease specific aspects were assessed in all patients.

Disease severity was measured by the Toronto Western Spasmodic Rating Scale (TWSTRS) and Cervical Dystonia Impact Profile (CDIP‐58), two internationally used rating scales which have been proven to be valid and reliable (Albanese et al., 2013). The TWSTRS score ranges from 0 to 85, the score on the CDIP‐58 ranges from 0 to 100 with a higher score indicating a more severe form of CD.

Dystonic head tremor was assessed by Tsui scale (Tsui, Eisen, & Calne, 1986) as this is not included in the TWSTRS nor the CDIP‐58. Severity of tremor is noted as mild = 1 or severe = 2. The duration is noted as occasional = 1 or continuous = 2. The tremor score = severity × duration and leads to a score from 0 to 4.

**Figure 1:**
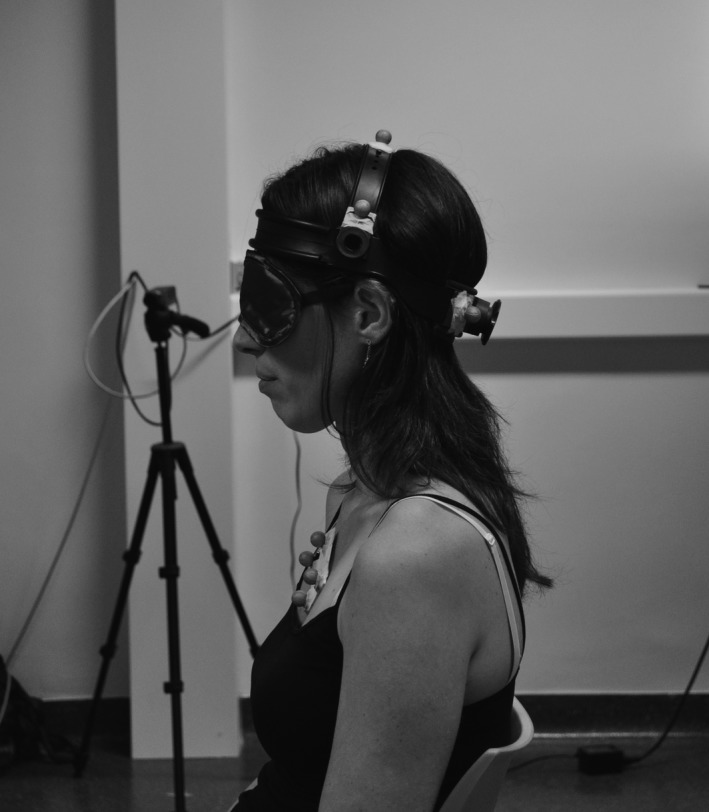
Placement of VICON ^®^ markers

Cervical sensorimotor control is evaluated by JPE in the head repositioning accuracy (HRA) test, which is measured in degrees (°). Measurements were obtained via 3D motion analysis using an infrared camera system with eight cameras recording at 100 Hz (VICON^®^ T10, Oxford Metrics, Oxford). Rigid plates with reflective markers were placed on the head and sternum (see Figure 1). No alleviating effect was reported of the pressure of the head band in the patient group. The measurement error of the VICON^®^ T10 system in Multidisciplinary Motor Centre Antwerp M^2^OCEAN is <1°(Sanders, Vereeck, & Hallemans, 2012). The measuring frequency was cut‐off at 10 Hz. In the HRA test, blindfolded participants have to relocate their head as accurately as possible to a self‐determined NHP after performing an active movement (flexion, extension, left and right rotation of the neck) (Revel et al., 1991). The NHP for patients was equal to the dystonic head position. Participants were seated on a chair with the arms resting on their lap, with their back against the back rest. They were asked to perform the neck movements within comfortable limits to avoid supplementary nociceptive input without using sensory tricks. This test was verbally explained, followed by a demonstration and performed 10 times by the participant for each plane of movement. To reduce the interference of fatigue, the order of testing was randomized by computer prior to testing. This test is proven to be valid and reliable (Michiels et al., 2013).

### 2.3 Data processing

The captured data of the Vicon^®^ markers were first reconstructed and labeled using Nexus^®^ software (version 1.8.5, RRID:SCR_015001). Afterwards, a custom‐made biomechanical model was used to calculate angle positions for each captured frame. Hence, movement angles of the neck were calculated using XYZ Euler/Cardan rotations of the head segment relative to the sternum segment. These data were then processed, using a custom made MATLAB^®^ code (version R2014a, MathWorks Inc., USA, RRID:SCR_001622) to calculate the neck angles.

The JPE can be calculated quantitatively by the absolute error (AE) or qualitatively by the constant error (CE) (Treleaven et al., 2003; Hill et al., 2009). The absolute error (AE) is the mean of the total deviation from the starting point over the trials (Treleaven et al., 2003).

$$\begin{array}{cc} & \text{AE}=(\text{absolute of raw error trial}\;1)+(\text{absolute of raw error trial}\;2)\\ & \;+\ldots +(\text{absolute of raw error trial}10)/10. \end{array}$$

The constant error (CE) is a measure of both direction and deviation from the starting point. It is calculated as the mean of the raw error over the trials incorporating the positive and negative values in each trial (Hill et al., 2009).

CE=(raw error trial1)+(raw error trial2)+…+(raw error trial10)/10.

When participants ‘undershoot’, i.e., fall behind the NHP, this results in a negative CE for flexion and left rotation and a positive CE for extension and right rotation. When participants ‘overshoot’, i.e., surpasses the NHP, this results in a positive CE for flexion and left rotation and in a negative CE for extension and right rotation.

A small repositioning error, represented by a small AE and a CE close to 0 represents good cervical sensorimotor control. In a population of patients with neck pain or whiplash‐associated disorders, the AE is 0.58°–1.66° larger than in controls, dependent on the measurement device (Kristjansson et al., 2003; Elsig et al., 2014; Wibault, Vaillant, Vuillerme, Dedering, & Peolsson, 2013).

### 2.4 Statistical methods

Data were analyzed using SPSS^®^ vs. 22 (RRID:SCR_002865). Normality of data was checked using the Kolmogorov–Smirnov test and level of significance was set at *p *<* *.05.

Intergroup differences were calculated by means of independent samples T‐test for normally distributed variables and a Mann–Whitney *U* test for nonnormally distributed variables. A post hoc Bonferroni correction was applied, given the multiple outcome measures for the HRA test. Analyzing the results of patients with and without dystonic head tremor showed no differences. Thus, separate analysis of patients with head tremor was considered to be unnecessary.

As sensory tricks alter sensorimotor processes, subdivision of the patient group was made based on the effect of sensory tricks. A Kruskall–Wallis test was performed to calculate intergroup differences.

Correlations between cervical sensorimotor control and the disease duration, severity of the disease as measured by the CDIP‐58, the total score and scores on the subscales of the TWSTRS were calculated using a Spearman rank correlation.

## 3 RESULTS

Kinematic assessments were performed on 24 patients with CD (20 females, 4 male, mean age 59.2 year ±13.9, range 30–86) and 70 control subjects (37 females, 33 males, mean age 54.5 year ±16.0, range 30–85). There were no significant differences in age between the patient and control group (*p *=* *.414). In 8.3% of the patients, the head posture was a pure torticollis. Other patients showed a combined form. All patients could move away from the dystonic posture. Table 1 presents patient characteristics such as side of dystonia, time elapsed since diagnosis of CD (disease duration), percentage of participants showing a dystonic head tremor, severity, and disability of CD (TWSTRS and CDIP‐58). No patients showed evidence of essential tremor.

**Table 1:** Characteristics of patient group (*N *=* *24)

	Mean	SD	Range
Mean disease duration (years) ±SD (range)	13.0	8.7	2–35
TWSTRS score	36.1	9.7	27.0–61.7
CDIP‐58 score	47.7	13.8	28.6–73.4
Symptoms: Head posture (%)			
Pure torticollis	8.3		
Pure laterocollis	4.2		
Torticollis and laterocollis	54.2		
Combined torticollis with anterocollis, retrocollis, or shift	33.3		
Presence of tremor (%)	45.8		

TWSTRS, Toronto Western Spasmodic Torticollis Rating Scale (maximum score of 85); CDIP‐58, Cervical Dystonia Impact Profile (maximum score of 100); SD, standard deviation.

Larger JPE was measured in patients with CD compared to controls for both the AE and CE (*p* ranges from .015 to <.0001) (see Table 2).

**Table 2:** Group comparison of joint position error of patients with cervical dystonia versus healthy controls (Mann–Whitney *U* test)

	Patients *n *=* *24	Controls *n *=* *70	*p*
	Mdn (IQR)	Mdn (IQR)	
Absolute error			
Extension	4.79° (6.20°)	3.06° (2.52°)	.015*
Flexion	4.66° (2.20°)	3.04° (2.60°)	.002**
Left rotation	3.69° (3.41°)	2.05° (1.20°)	<.0001**
Right rotation	3.37° (2.58°)	2.22° (1.71°)	.006**
Constant error			
Extension	−2.93° (4.70°)	1.07° (4.11°)	<.0001**
Flexion	3.39° (2.62°)	−1.29° (3.68°)	<.0001**
Left rotation	2.33° (5.31°)	−0.25° (2.33°)	.001**
Right rotation	−1.21° (3.24°)	0.45° (3.61°)	.004**

Mdn, Median; IQR, interquartile range.

*Significant at 0.05 level, **significant after Bonferroni correction *p* < .00625 (.05/8 = .00625).

**Figure 2:**
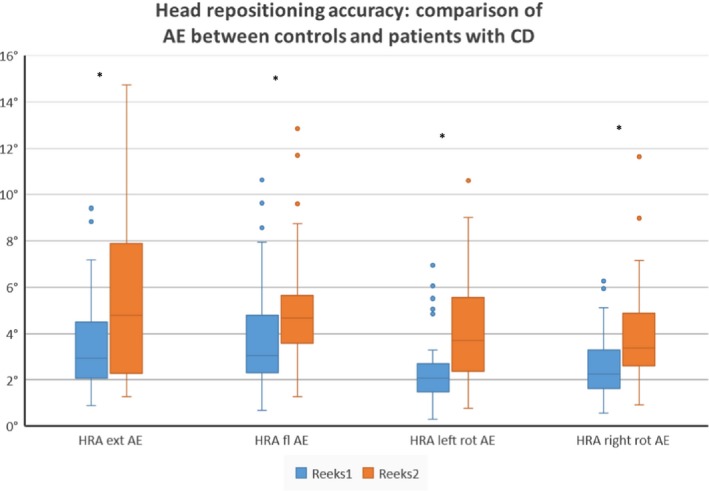
Intergroup differences between controls and subject with CD for the absolute error in the head repositioning accuracy test. Boxplots represent median and interquartile range of the absolute error (AE) measured during the head repositioning accuracy test (HRA) after performing extension, flexion, left rotation, and right rotation (*significant at *p *<* *.05 level)

The AE in the patient group is 1.73° larger after performing an extension movement compared to the control group, 1.62° larger after flexion, 1.64° after left rotation and 1.15° after performing a right rotation movement (see Table 2 and Figure 2).

**Figure 3:**
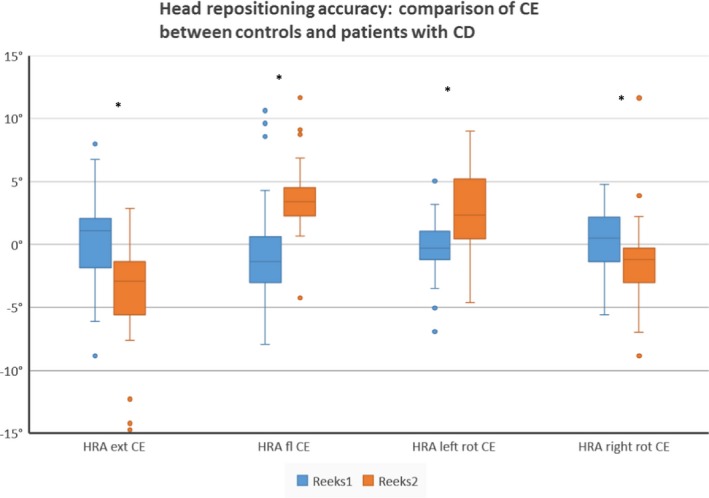
Intergroup differences between controls and subject with CD for the constant error in the head repositioning accuracy test. Boxplots represent median and interquartile range of the constant error (CE) measured during the head repositioning accuracy test (HRA) after performing extension, flexion, left rotation, and right rotation (*significant at *p *<* *.05 level)

The CE in the patient group is larger than in the control group (*p* ranges from .004 to <.0001). Moreover, healthy subjects undershoot to the NHP, where patients with CD overshoot to the NHP in every movement direction (see Table 2 and Figure 3). This opposite motor behavior is seen in 75% of the patients. The larger JPE is not side specific nor correlated with the side of dystonic posture.

A subdivision of the patient group based on the perceived effect of sensory tricks showed in two parameters that patients who experience no benefit of sensory tricks tend to have a smaller JPE than patients who have partial or complete relief of sensory tricks. Both parameters (AE extension and CE flexion) are in the sagittal plane of movement and are not significant after a Bonferroni correction (*p *>* *.00625).

No correlations were found between both the AE, CE, (both measures of the JPE), and the clinical characteristics disease duration, head tremor, age, disease severity (as measured by the total scores on the CDIP‐58 and TWSTRS), and the subscales of the TWSTRS (severity, disability and pain subscale).

## 4 DISCUSSION

The aim of this cross‐sectional study was to investigate deficiencies in cervical sensorimotor control in patients with adult‐onset idiopathic CD by evaluating the JPE in a head repositioning task in which visual input is eliminated. The main finding is that the JPE in repositioning to a neutral head position is larger in patients with CD, reflecting an impaired cervical sensorimotor control. Additionally, patients with CD systematically overshoot beyond the neutral head position.

Several reports documented affected somatosensory dysfunctions in CD. Abnormalities in proprioception in distant areas from the neck have been shown (Grünewald et al., 1997; Putzki et al., 2006), whereas research concerning neck proprioception reported contradicting results. Bove et al. (Bove et al., 2004; Bove, Courtine, & Schieppati, 2002) reported different responses of patients with CD to muscle vibration on the sternocleidomastoid muscles. Some patients seemed to ignore this altered information, while others showed opposite reactions compared to controls. Anastasopoulos et al. first reported normal kinesthetic perception of the neck (Anastasopoulos, Nasios, Mergner, & Maurer, 2003). More recently, however, they reported an impaired voluntary suppression of cervicocollic reflex, leading to the conclusion that sensory driven stabilization of the head is impaired in CD (Anastasopoulos et al., 2014). Our results add to the body of literature showing that CD is associated with somatosensory dysfunctions during voluntary head movements. To distinguish between a sensory or motor deficit, further studies are needed.

The larger JPE in our sample is comparable to impaired cervical sensorimotor control in chronic neck pain patients and patients with whiplash‐associated disorders (Revel et al., 1991; Treleaven et al., 2003; Michiels et al., 2013; Elsig et al., 2014; Wibault et al., 2013). Subdividing our patient group showed that patients who benefit from sensory tricks or alleviation maneuvers tend to have larger joint repositioning errors than patients who do not benefit from sensory tricks. Sensory tricks alter sensorimotor processes and are an indicator of the sensory component in cervical dystonia (Patel et al., 2014). Patients benefitting from sensory tricks tend to show larger impairments in cervical sensorimotor control. The difference is, however, not significant. The data of the total patient population show that the JPE is not only larger, patients show opposite motor control compared to healthy controls. In healthy controls, the repositioning error varies around 0° with a small constant error. The patient group, however, systematically overshoots and surpasses the NHP, e.g., the dystonic posture in patients with CD, in every movement direction, whereas control subjects undershoot to the NHP. The overshoot is apparent in every plane of movement and is present only on the dystonic side. We therefore assume that the diminished cervical sensorimotor control is a characteristic inherent to CD.

Researchers previously attributed the overshoot of patients with neck pain to a dysfunction in sensory information from the cervical muscle spindles where patients overshoot in a search for additional proprioceptive input (Revel et al., 1991; Treleaven et al., 2008). The origin of the impaired cervical sensorimotor control in patients with CD could be attributed not only to deficiencies in peripheral sensory input but also to impaired central integration and processing. In sensorimotor control, sensory afferent input is integrated by the central nervous system and used for constructing an appropriate motor response (Konczak & Abbruzzese, 2013). Peripheral sensory input, as in neck proprioception, could indeed be impaired by altered afferent muscle spindle information. Disorders in afferent muscle spindle processing have been reported not only in the affected region but also in sites remote from the neck in patients with CD (Bove et al., 2004; Lekhel et al., 1997; Grünewald et al., 1997). Next to peripheral input, central neural integration of different network input is essential and impairments have been reported (Tinazzi et al., 2009; Molloy et al., 2003; Zoons, Booij, Nederveen, Dijk, & Tijssen, 2011; Neychev, Gross, Lehéricy, Hess, & Jinnah, 2011; Filip, Lungu, Shaw, Kasparek, & Bareš, 2013) such as higher spatial and temporal somatosensory discrimination thresholds in adult‐onset focal dystonia (Tinazzi et al., 2009; Molloy et al., 2003). As multiple brain regions are involved in the pathology of CD, network models are presented (Shaikh, Zee, Crawford, & Jinnah, 2016; Prudente, Hess, & Jinnah, 2014) with recent evidence for cerebellar involvement in the pathophysiology of CD (Neychev et al., 2011; Filip et al., 2013; Shaikh et al., 2016; Malone, Manto, & Hass, 2014; LeDoux & Brand, 2003). Maintaining posture, originating and coordinating voluntary movement are some of the functionalities of the cerebellum. The cerebellum is also a direct recipient of sensory input due to the representation of the somatosensory cortex in the cerebellar cortex (Manto et al., 2012). This leads to the hypothesis that cerebellar mechanisms could explain the impaired cervical sensorimotor control. Using the HRA test, dysfunctions in cervical sensorimotor control can be evaluated; however, we cannot differentiate whether this is a result of impaired cerebellar, basal ganglia or peripheral feedback or central processing. Further research may clarify which mechanism is responsible for the impaired sensorimotor control.

Potential sources of bias could relate to the experimental setup. First, the presence of pain could influence the HRA test (Eva‐Maj et al., 2013). To prevent supplementary nociceptive input arising from the neck, patients were instructed to perform the active movement without additional pain provocation. Additionally, no correlation was found between the pain intensity and the JPE. Therefore, we believe that pain during the test did not affect the JPE. Secondly, vestibular input could influence the HRA test. When moving one's head at a velocity of more than 2.1°/s, vestibular input increases and cervical afferent input decreases (Kelders et al., 2003). In our sample, patients with vestibular symptoms were excluded. We therefore believe vestibular input was not responsible for the altered cervical sensorimotor control. Thirdly, since no gender differences have been reported in literature (Artz, Adams, & Dolan, 2015; de Vries et al., 2015), nor been found in our normative database, we do not believe gender affected our results.

Assessing cervical sensorimotor control by the HRA test can easily be conducted in clinical practice. The JPE can be measured in a valid and reliable way by conducting a head repositioning task using a laser pointer (Michiels et al., 2013). The affected cervical sensorimotor control in patients with CD can be addressed in treatment. Training cervical sensorimotor control in patients with neck pain or whiplash‐associated disorders has been proven to alleviate neck complaints (Revel et al., 1994; Jull et al., 2006; Chiarotto et al., 2015). A multimodal physical therapy program additional to botulinum toxin has a positive effect on pain, head posture, and functioning in everyday life activities (De Pauw et al., 2014). Incorporating cervical sensorimotor control exercises in physical therapy programs could potentially help patients with CD performing voluntary movements and keep their head upright, although further studies are required to assess the efficacy.

## CONFLICT OF INTEREST

No conflict of interest is to be reported.
